# Accumulated ROS Activates HIF-1α-Induced Glycolysis and Exerts a Protective Effect on Sensory Hair Cells Against Noise-Induced Damage

**DOI:** 10.3389/fmolb.2021.806650

**Published:** 2022-01-12

**Authors:** Shuo Liang, Shuohui Dong, Wenwen Liu, Man Wang, Shanshan Tian, Yu Ai, Haibo Wang

**Affiliations:** ^1^ Department of Otolaryngology-Head and Neck Surgery, Shandong Provincial ENT Hospital, Cheeloo College of Medicine, Shandong University, Jinan, China; ^2^ Department of General Surgery, Shandong Qianfoshan Hospital, Cheeloo College of Medicine, Shandong University, Jinan, China

**Keywords:** sensory hair cells, noise-induced hearing loss, oxidative stress, glycolysis, HIF-1α, mitochondria, energy metabolism

## Abstract

Noise exposure causes noise-induced hearing loss (NIHL). NIHL exhibits loss of inner ear sensory hair cells and is often irreparable. Although oxidative stress is involved in hearing loss, the complex mechanisms involved in NIHL are unclear. Hypoxia-inducible factor 1α (HIF-1α) has been suggested to be essential for protecting sensory hair cells. Additionally, it has been shown that ROS is involved in modulating the stability of HIF-1α. To investigate the NIHL pathogenesis, we established a tert-butyl hydroperoxide (t-BHP)-induced oxidative stress damage model in hair-like HEI-OC1 cells and an NIHL model in C57BL/6 mice. Protein and mRNA expression were determined, and biochemical parameters including reactive oxygen species (ROS) accumulation, glucose uptake, adenosine triphosphat (ATP) production, and mitochondrial content were evaluated. In HEI-OC1 cells, t-BHP induced ROS accumulation and reduced mitochondrial content and oxygen consumption, but the ATP level was unaffected. Additionally, there was increased glucose uptake and lactate release along with elevated expression of HIF-1α, glucose transporter 1, and several glycolytic enzymes. Consistently, noise trauma induced oxidative stress and the expression of HIF-1α and glycolytic enzymes in mice. Thus, we concluded that ROS induced HIF-1α expression, which promoted glycolysis, suggesting a metabolic shift maintained the ATP level to attenuate hair cell damage in NIHL.

## 1 Introduction

Noise-induced hearing loss (NIHL) is a progressive reduction in hearing ability or even hearing loss caused by noisy environment exposure (Wang and Puel 2018; Chadha et al., 2021). NIHL is the most important cause of acquired hearing loss that underlies 16% of sensorineural hearing loss in adults worldwide, which has always been a significant public health issue globally (Chadha et al., 2021). Specific damage during NIHL includes hearing threshold elevation and loss of sensory hair cells in the inner ear (Henderson et al., 2006; Cunningham and Tucci 2015). Sensory hair cells are the receptors of the inner ear that are critical for the hearing ability (Wu et al., 2019). As terminally differentiated sensory cells, the destruction and loss of hair cells is often irreparable (Shrestha et al., 2011). Audiological features and cochlear morphology of NIHL are well characterized, but pathomechanisms underlying NIHL are complex and not completely understood. Although several theories have been attempted to explain the occurrence of NIHL using reasons, including genetic susceptibility, oxidative stress injury, inflammatory reactions, and immune responses (Henderson et al., 2006; Kurabi et al., 2017), there is no consensus on the exact mechanism. Thus, in-depth studies to develop comprehensive understandings of the pathogenesis of NIHL is urgently needed.

Oxidative stress injury is a well-recognized element of the pathogenesis of hearing loss (Henderson et al., 2006; Sha and Schacht 2017; Fetoni et al., 2019). Oxidative stress injury is attributed to an imbalance between the production and removal of reactive oxygen species (ROS) (Guo et al., 2017). Abnormal accumulation of intracellular ROS may contribute to a multitude of pathological conditions. Several studies have shown that exposure to traumatic noise caused structural destruction of the inner ear by accumulation of excessive ROS (Henderson et al., 2006; Zorov et al., 2012; Fetoni et al., 2019). In specific processes, ROS activates cascade reactions to degrade certain cochlear cells, especially sensory hair cell dysfunction and eventually death (Sha and Schacht 2017; Zhang et al., 2019). The negative role of excessive ROS in NIHL appears to be accepted. Nevertheless, we noticed that several studies about NIHL have reported that ROS-dependent signaling molecules also have positive effects on adaptation to stress and cell survival in excessive ROS conditions (Leslie 2006; Collins et al., 2012; Sena and Chandel 2012). The discrepancies between these theories may be explained by the fact that ROS, as a double-edged sword, has dual roles during the initiation, promotion, and progression stages of NIHL. In the current study, we explored the mechanisms of the effects of ROS-dependent signaling molecules on the pathophysiology of NIHL.

Hypoxia-inducible factor 1α (HIF-1α) is highly inducible under hypoxic conditions and functions as a prime transcription factor in mRNA production (Wang et al., 1995). Under normoxia, HIF-1α is hydroxylated on its proline residues by prolyl hydroxylases (PHDs), resulting in its ubiquitination and rapid degradation by proteasomes (Harris 2002). Several studies have shown that ROS, which act as negative regulators on PHDs’ activity and play the same role as the hypoxia, stabilized HIF-1α by inhibiting its ubiquitin degradation, which led to its accumulation and active gene expression (Mansfield et al., 2005; Li et al., 2014; Chen et al., 2018). HIF-1α as a transcription factor that can be regulated, activates many oxygen-sensitive genes such as those encoding erythropoietin, vascular endothelial growth factor, glucose transporters, glycolytic enzymes, inducible nitric oxide synthase, and hemeoxygenase-1 (Liu et al., 2002). The transcription products of some of these oxygen-sensitive genes play a beneficial role in regulating glucose transport and glycolysis, thereby modifying cellular energy metabolism pathways (Halligan et al., 2016). Recent studies have indicated that HIF-1α plays a potential role in preventing hearing loss and protecting sensory hair cells in NIHL (Chung et al., 2004; Chung et al., 2011), while the underlying mechanisms are unclear.

Sensory hair cells are highly sensitive receptors that mediate hearing and have high energy requirements (Kucharava et al., 2019). They contain a large number of mitochondria, “energy factories” of the cell, to accommodate their high metabolic load (O'Reilly et al., 2019), and their main source of energy is mitochondrial oxidative phosphorylation (McPherson 2018). Several studies have reported that ROS-mediated mitochondrial respiratory dysfunction and insufficient energy supply can cause ROS-induced sensory hair cell injuries (Kwon et al., 2015; O'Reilly et al., 2019). Furthermore, although normal sensory hair cells do not utilize glycolysis as their preferential energy source, impaired glycolysis has been shown to promote apoptosis in sensory hair cells (Kang et al., 2020). Therefore, energy metabolism homeostasis is essential for normal of sensory hair cell function. Based on the current research, we argue that the promotion of glycolysis following ROS-induced HIF-1α activation is an essential metabolic remodeling event in sensory hair cells under oxidative stress, and that increased glycolysis may exert a protective effect on sensory hair cells against noise-induced damage.

## 2 Materials and Methods

### 2.1 Reagents and Antibodies

We purchased a cell counting kit-8 (CCK8) (C0037), a mitochondria isolation kit (C3601), a BCA protein assay kit (P0012S), a ROS assay kit (S0033S), and an ATP assay kit (S0026) from Beyotime, China. We also purchased 2-NBDG (HY-116215) from MedChemExpress, China, 3-bromopyruvatic acid (3-BrPA) (S5426) and AZ-33 (S0108) from Selleck, China. A lactate release assay kit (KGT023) was purchased from KeyGEN, China. We also purchased a superoxide dismutase assay kit (ab65354), a glutathione peroxidase assay kit (ab102530), a catalase activity assay kit (ab83464), a lactate dehydrogenase activity assay kit (ab102526), and a mitochondrial staining kit (ab112145) from Abcam, United States. The following antibodies were used: anti-SOD1 (rabbit monoclonal, ab51254, Abcam), anti-SOD2 (rabbit monoclonal, ab68155, Abcam), anti-Catalase (rabbit polyclonal, 21260-1-AP, Proteintech), anti-GPx1 (rabbit monoclonal, ab108429, Abcam), anti-GLUT1 (mouse monoclonal, ab238050, Abcam), anti-HK2 (rabbit monoclonal, ab209847, Abcam), anti-ENO1 (rabbit monoclonal, ab227978, Abcam), anti-PDK1 (rabbit monoclonal, ab202468, Abcam), anti-LDHA (mouse monoclonal, 66287-1-Ig, Proteintech), anti-HIF-1α (rabbit monoclonal, ab179483, Abcam; mouse monoclonal, 66730-1-Ig, Proteintech), anti-β-Tubulin (rabbit polyclonal, 10068-1-AP, Proteintech), anti-Myosin VIIa (rabbit monoclonal, ab155984, Abcam), and 4-HNE (mouse monoclonal, ab48506, Abcam).

### 2.2 Cells Culture

House Ear Institute-Organ of Corti 1 (HEI-OC1) cells derive from the auditory organ of the transgenic mouse Immortomouse™. HEI-OC1 cells are the most potent *in vitro* model for sensory hair cells and express specific molecular markers of sensory hair cells, including Myosin VIIa. HEI-OC1 cells were cultured in Dulbecco’s modified Eagle’s medium (DMEM, high glucose) supplemented with 10% (v/v) fetal bovine serum. Cells were passaged every 3 days using 0.25% trypsin-EDTA for dissociation. Cells were incubated in a 33°C 10% CO_2_ humidified incubator. For *Hif1a* knockdown, we used Lipo2000 to transfect HEI-OC1 cells with siRNA targeting *Hif1a* (5′-GGA​AAG​AAC​TAA​ACA​CAC​A-3′) (Jikai Gene, China) or a negative control (Jikai Gene, China).

### 2.3 Animal

C57 BL/6 mice (male, 6 weeks-of-age) were purchased from Shandong University Experimental Animal Center (Jinan, China) and housed in the animal center of Shandong Provincial ENT Hospital affiliated to Shandong University. All procedures were approved by the Institutional Animal Care and Use Committee of Shandong Provincial ENT Hospital affiliated to Shandong University. All animal studies complied with relevant ethical regulations for animal testing and research (Ethics approval number: No. XYK20210212).

### 2.4 CCK8 Cytotoxicity Assay

To detect the influence of t-BHP on HEI-OC1 cells. HEI-OC1 cells (1 × 10^4^ cells per well) were seeded into 96-well plates and grown overnight. Adherent HEI-OC1 cells were treated with different concentrations (0, 20, 40, 60, 80, 100, 120, 140, 160, 180, and 200 μM) of t-BHP for 4 h. To determine the cytotoxic effect of glycolytic inhibitors on HEI-OC1 cells. 4 × 10^3^ HEI-OC1 cells per well were seeded into 96-well plates and grown overnight. HEI-OC1 cells were cultured with or without 80 µM t-BHP for 4 h, and then treated with indicated inhibitors for 48 h. After treatment, the medium was carefully removed and cells were washed with ice-cold PBS. Then, 10% (v/v) of WST-8 dye (CCK8 Kit, Beyotime, China) was added and incubated for 1 h in a 33°C 10% CO_2_ humidified incubator. At the end of incubation, the absorbance was recorded at 450 nm using a microplate reader (Bio-Rad, CA, United States) after mixing gently on an orbital shaker for 1 min. Cell viability was calculated as follows: (absorbance of sample - absorbance of blank)/(absorbance of control - absorbance of blank) × 100%. All experiments were performed with five replicates.

### 2.5 BCA Protein Assay

Protein concentrations were quantified using a BCA protein assay kit (Beyotime, China). Briefly, 20 μl supernatant containing the proteins was mixed with 200 μl BCA working solution in 96-well plates. After incubation at 37°C for 30 min, the absorbance was recorded at 595 nm using a microplate reader, and protein concentration was determined based on the standard curve. All experiments were performed with at least three replicates.

### 2.6 Mitochondria Isolation

HEI-OC1 cells were seeded at a density of 5 × 10^4^ per 15-cm culture dish, grown to 80–90% confluency in a humidified incubator at 33°C 10% CO_2_ and then treated with 80 µM t-BHP for 4 h or left untreated. After trypsin digestion and centrifugation, mitochondria were isolated using a mitochondria isolation kit (Beyotime, China). Briefly, cells were homogenized and centrifuged at 600 × g for 10 min. Supernatants were centrifuged again at 11,000 × g for 10 min to obtain purified mitochondria in the precipitate. Isolated mitochondria were lysed with assay buffer of lactate dehydrogenase (LDH) activity assay kit and used to detect mitochondrial LDH activity.

### 2.7 Superoxide Dismutase Activity Assay

2 × 10^6^ untreated HEI-OC1 cells or those treated with 80 µM t-BHP were collected and lysed with ice-cold cell lysis reagent (0.1 M Tris/HCl, pH 7.4, containing 0.5% Triton X-100, 5 mM β-ME, 0.1 mg/ml phenylmethylsulfonyl fluoride). Following centrifugation at 14,000 × g for 5 min, supernatants were collected and used for assays. SOD activity was measured using a colorimetric SOD activity assay kit (Abcam, United States) in 96-well plates. Briefly, 20 µl of the sample was mixed with WST working solution and enzyme working solution, and activities of SOD were calculated by measuring the absorbance at 450 nm following incubation at 33°C for 20 min. All experiments were performed in five replicates.

### 2.8 Glutathione Peroxidase Activity Assay

2 × 10^6^ indicated HEI-OC1 cells were harvested after t-BHP treatment and GPx activity assays were performed using the colorimetric GPx activity assay kit (Abcam, United States). The collected cells were washed with ice-cold PBS and were lysed with 200 µl cold Assay Buffer. Following centrifugation 15 min at 4°C at 10,000 × g using a cold microcentrifuge to remove any insoluble material, the supernatants were transferred to clean tubes. Then, 10 µl sample was added to the mixture reaction system (40 mM NADPH solution, glutahione reductase, and glutathione) in 96-well plates. After incubating at room temperature for 15 min, 10 µl cumene hydroperoxide solution was added to reaction system. The concentration of NADPH was measured at 340 nm using a microplate reader at 0 and 5 min after cumene hydroperoxide addition, and GPx activities were calculated from the changes of NADPH concentrations. All experiments were performed with five replicates.

### 2.9 Catalase Activity Assay

1 × 10^6^ indicated HEI-OC1 cells with or without 80 µM t-BHP treatment were collected, and CAT activity assays were performed using the colorimetric CAT activity assay kit (Abcam, ab83464). Washed Cells were resuspended in ice-cold assay buffer and repeatedly blown with a pipette gun. Samples were then centrifuged 15 min at 4°C at 10,000 × g and supernatant removed. 10 µl sample was mixed with 12 µl of fresh 1 mM H_2_O_2_ solution and 68 µl assay buffer in 96-well plates. After incubating at room temperature for 30 min, 10 µl stop solution was added to each sample.

Then, 50 µl of Developer Mix (containing OxiRed probe and HRP solution) was added to each reaction, and plates were avoided light and incubated at room temperature for 10 min. Finally, the amount of H_2_O_2_ was measured at 570 nm on a microplate reader, and CAT activities were calculated from the consumption of H_2_O_2._ All experiments were performed with five replicates.

### 2.10 Lactate Dehydrogenase Activity Assay

Indicated HEI-OC1 cells treated with or without 80 µM t-BHP for 4 h. Then the 2 × 10^6^ cells were collected and lysed with ice-cold assay buffer. Following centrifugation at 10,000 × g for 15 min, the supernatants were obtained and then used for assays. Then, LDH activity was measured with the colorimetric LDH activity assay kit (Abcam, United States). 10 µl sample was mixed with 40 µl assay buffer and 50 µl reaction mix in 96-well plates. After incubating at 33°C for 15 min, the activities of LDH were calculated by measuring the absorbance at 450 nm. All experiments were performed with five replicates.

### 2.11 Mitochondrial Content Assay

Indicated HEI-OC1 cells (2 × 10^5^ cells per well) were seeded into 6-well plates and grown to 80% confluency, and then cells were treated with or without 80 µM t-BHP for 4 h. After treatment, HEI-OC1 cells were cultured with fluorescent Mito-Tracker (a hydrophobic compound, easily permeates intact live cells and becomes trapped in mitochondria after it gets into cells) (Abcam, United States) for 1 h in a 33°C 10% CO_2_ humidified incubator. At the end of incubation, the cells were digested and kept on ice protected from light. The fluorescence arising from Mito-Tracker (Texas Red channel) was then detected by a BD FACSAria II instrument (BD, United States). A total of 20,000 events were collected per sample, and polygonal gating was used to exclude debris. Data were analyzed with FlowJo Software. All experiments were performed with five replicates.

### 2.12 Intracellular Reactive Oxygen Species Assay

When indicated HEI-OC1 cells in 6-well plates had reached 80% confluence, they were treated with or without 80 µM t-BHP. After 4 h, adherent cells were washed with PBS and fresh media containing 10 μM 2′,7′-dichlorodihydrofluorescein diacetate (DCFH-DA) probes (Beyotime, China) was added. Cells were cultured with probes for 30 min in a 33°C 10% CO_2_ humidified incubator. Then, the cells were washed with ice-cold PBS and digested with trypsin-EDTA, and kept on the ice protected from light. The fluorescence of DCFH-DA (FITC channel) was detected by a BD FACSAria II instrument (BD, USA). 20,000 events from each specimen were measured to pledge sufficient data. Data were analyzed with FlowJo Software and polygonal gating was used to exclude debris. All experiments were performed with five replicates.

### 2.13 ATP Production Assay

2 × 10^5^ indicated HEI-OC1 cells per well were seeded into 6-well plates until 80% confluence was achieved. Cells were treated with or without 80 µM t-BHP for 4 h, and then cells were lysed with lysis buffer and centrifuged at 12,000 × g for 5 min at 4°C. Fresh whole-cell lysates used for detecting ATP (Beyotime, China) and total protein concentrations. The supernatants were mixed with detection solution and then analyse for ATP concentrations with a luminometer and determined based on the standard curve. ATP levels were normalized to total protein concentrations (BCA assays). All experiments were performed with five replicates.

### 2.14 2-NBDG Uptake Assay

2 × 10^5^ indicated HEI-OC1 cells were seeded per well in 6-well plates, grown to 80% confluence in a 33°C 10% CO_2_ humidified incubator and then treated or without 80 µM t-BHP for 4 h. Cells were incubated with sugar-free DMEM medium with 100 μM fluorescent glucose analog 2-NBDG (MCE, China) for 2 h in an incubator. Then, the cells were digested and kept on ice protected from light. The fluorescence arising from 2-NBDG (FITC channel) was then detected by a BD FACSAria II instrument (BD, United States). 20,000 events from each specimen were measured to pledge sufficient data. Data were analyzed with FlowJo Software and polygonal gating was used to exclude debris. All experiments were performed with five replicates.

### 2.15 Lactate Release Assay

2 × 10^4^ indicated HEI-OC1 cells per well were seeded into 24-well plates. When cells had reached 60% confluence, the medium was replaced with 1 ml fresh complete medium per well and supernatants were collected after 24 h. The concentration of lactate in supernatant was determined with a lactate assay kit (KeyGEN, China). Briefly, 20 µl supernatant was mixed with the reaction system in 5 ml centrifuge tube. Centrifuge tubes were incubated in a waterbath at 37°C for 10  min, and then 2 ml stop solution was added to reaction system immediately. Finally, the absorbance of the colored substance was measured at 530 nm, and the OD values had a linear relationship with the content of lactate. In particular, OD values of samples between 0.05 and 0.35 are acceptable according to the manufacturer’s protocol of the lactate assay kit. Additionally, protein quantitation was measured by the BCA assays and lactate release counts were normalized to total protein concentrations (mmol/d/g protein). All experiments were performed with five replicates.

### 2.16 Measurement of Oxygen Consumption Rates and Extracellular Acidification Rates

Agilent Seahorse XFe96 Analyzer (Agilent, United States) was used to measure the OCR and ECAR of HEI-OC1 cells in 96-well plates. OCR and ECAR are key indicators of mitochondrial respiration and glycolysis, and together these measurements provide a system-level view of cellular metabolic function in cells. Specifically, 1 × 10^4^ HEI-OC1 cells were seeded in an XFe96 cell culture 96-well plate and incubated overnight in a humidified incubator at 33°C with 10% CO_2_. To equilibrate the temperature and pH of the detection system, cells were washed with assay DMEM medium and incubated in a CO_2_-free incubator for 1 h before assessment. OCR and ECAR were measured with an Agilent Seahorse XFe96 Analyzer using template-matching procedures. All experiments were performed in five replicates.

For mitochondrial respiration assessment, cells were treated with 1.5 µM oligomycin, 1 µM carbonyl cyanide p-trifluoromethoxyphenylhydrazone (FCCP), and 0.5 µM rotenone/antimycin A using a Seahorse XF Cell Mito Stress Test Kit (Agilent, United States). Initially, mitochondrial basal respiration was determined at the baseline of the OCR. The first injection was 1.5 µM oligomycin (an ATP synthase/complex V inhibitor), which was linked to mitochondrial ATP production. After 1 µM FCCP (an uncoupling agent that collapses the proton gradient and disrupts the mitochondrial membrane potential) was added, electron flow through the ETC was uninhibited and oxygen consumption by complex IV reached the maximum. The OCR at this point could be defined as maximal respiration. The final injection was a 0.5 µM mixture of rotenone (a complex I inhibitor) and antimycin A (a complex III inhibitor) that completely shut down mitochondrial respiration and enabled the calculation of non-mitochondrial respiration. Furthermore, several parameters of the OCR were calculated. Non-mitochondrial oxygen consumption was determined based on the OCR after the addition of rotenone/antimycin A. The basal mitochondrial rate was assessed as the OCR at baseline minus non-mitochondrial oxygen consumption. Mitochondrial ATP production was determined as the baseline OCR minus OCR after oligomycin addition. Maximal mitochondrial respiration was calculated as OCR after FCCP addition minus non-mitochondrial oxygen consumption (Figure 2I).

For assessment of glycolytic activity, cells were treated with 100 mM glucose, 10 μM oligomycin, and 500 mM 2-deoxy-D-glucose using a Seahorse XF glycolytic rate assay kit (Agilent, USA) First, HEI-OC1 cells were incubated in a medium without glucose or pyruvate, and the ECAR was measured, which was referred to as non-glycolytic acidification. The first injection was a saturation concentration of glucose (10 mM), and this glucose-induced response indicated the rate of glycolysis under basal conditions. The second injection was 10 μM oligomycin, which inhibited mitochondrial ATP production and shifted ATP production to glycolysis, with a subsequent increase in ECAR, revealing the maximum glycolytic capacity. The final injection was a 50 mM 2-DG (a glucose analog), which restricted glycolysis through competitive inhibition with glucose hexokinase. The resulting decrease in ECAR confirmed that the ECAR produced in the experiment was due to glycolysis. Furthermore, several parameters of the ECAR were calculated. Non-glycolytic acidification was assessed as ECAR at baseline without glucose injection. Basal glycolysis was calculated as ECAR after glucose addition minus non-glycolytic acidification. Glycolytic capacity was determined as the ECAR after oligomycin injection minus non-glycolytic acidification. Glycolytic reserve was calculated as the ECAR after 2-DG injection minus the ECAR after glucose injection (Figure 2K).

### 2.17 Western Blot Analysis

HEI-OC1 cells were cultured and treated in 6-well plates or T25 flasks, and cells were harvested when confluence reached 80–90%. Plates or flasks were washed three times with ice-cold PBS, and cells were lysed in RIPA buffer (KeyGEN, China) with a protease inhibitor (KeyGEN, China) on ice for 30 min. Lysates were then centrifuged at 14,000 × g for 20 min, and the supernatant was collected. The concentration of total protein was measured using a BCA protein assay kit (Beyotime, China). Next, the protein was boiled for 10 min with 5 × loading buffer (KeyGEN, China). Protein solutions were cooled on ice and subjected to SDS-PAGE (Bio-Rad, United States) electrophoresis. Proteins were transferred to 0.45 μm PVDF membranes (Millipore, Ireland) after electrophoresis. The membranes were blocked with 5% skim milk powder (Beyotime, China), and then incubated overnight at 4°C with primary antibodies. Antibodies used in the study were: anti-SOD1 (1:10000, Abcam), anti-SOD2 (1:1000, Abcam), anti-Catalase (1:2000, Proteintech), anti-GPx1 (1:2000, Abcam), anti-GLUT1 (1:5000, Abcam), anti-HK2 (1:1000, Abcam), anti-ENO1 (1:1000, Abcam), anti-PDK1 (1:1000, Abcam), anti-LDHA (1:1000, Proteintech), anti-HIF-1α (1:500, Abcam), and anti-β-Tubulin (1:5000, Proteintech). The second antibody was incubated for 1.5 h at room temperature. Proteins were detected by chemiluminescence using an Amersham Imager 600 (GE, United States). Each experiment was repeated three independent times.

### 2.18 Gene Expression Analysis

Total RNA was extracted with TRIzol reagent (TaKaRa, Japan) and the RNA concentration was determined with a NanoDrop spectrophotometer (NanoDrop Technologies, United States). RNA was then reverse transcribed into cDNA with a ReverTra Ace qPCR RT Kit (TOYOBO, Japan). The expression levels of target genes were then amplified by real-time quantitative polymerase chain reaction (RT-qPCR) with a SYBR Green Realtime PCR Master Mix (TOYOBO, Japan), gene-specific primers (Table 1), and a LightCycler 480 II instrument (Roche, Switzerland). The housekeeping gene *Tubb3* was used as an internal reference, and mRNA levels for each target were then calculated by the 2^−ΔΔ^Ct method. All experiments were performed with five replicates.

**TABLE 1:** Gene-specific primers in this study.

Gene	Forward primer (5–3′)	Reverse primer (5–3′)
*Sod1*	GGT​GAA​CCA​GTT​GTG​TTG​TCA​GG	ATG​AGG​TCC​TGC​ACT​GGT​ACA​G
*Sod1*	GGT​GAA​CCA​GTT​GTG​TTG​TCA​GG	ATG​AGG​TCC​TGC​ACT​GGT​ACA​G
*Sod2*	TAA​CGC​GCA​GAT​CAT​GCA​GCT​G	AGG​CTG​AAG​AGC​GAC​CTG​AGT​T
*Catalase*	CCT​CGT​TCA​GGA​TGT​GGT​TT	TCT​GGT​GAT​ATC​GTG​GGT​GA
*Gpx1*	CGC​TCT​TTA​CCT​TCC​TGC​GGA​A	AGT​TCC​AGG​CAA​TGT​CGT​TGC​G
*Glut1*	GCT​TCT​CCA​ACT​GGA​CCT​CAA​AC	ACG​AGG​AGC​ACC​GTG​AAG​ATG​A
*Hk2*	CCC​TGT​GAA​GAT​GTT​GCC​CAC​T	CCT​TCG​CTT​GCC​ATT​ACG​CAC​G
*Eno1*	TAC​CGC​CAC​ATT​GCT​GAC​TTG​G	GCT​TGT​TGC​CAG​CAT​GAG​AAC​C
*Pdk1*	CCA​CTG​AGG​AAG​ATC​GAC​AGA​C	AGA​GGC​GTG​ATA​TGG​GCA​ATC​C
*Ldha*	ACG​CAG​ACA​AGG​AGC​AGT​GGA​A	ATG​CTC​TCA​GCC​AAG​TCT​GCC​A
*Tubb3*	CAT​CAG​CGA​TGA​GCA​CGG​CAT​A	GGT​TCC​AAG​TCC​ACC​AGA​ATG​G

### 2.19 Immunocytofluorescence

1×10^5^ HEI-OC1 cells were seeded on cell climbing slices coated with poly-D-lysine in 24 well plates. When confluence was close to 60–80%, cell climbing slices were fixed with 4% paraformaldehyde for 20 min, permeabilized with 1% Triton X-100 for 10 min and then blocked in 10% goat serum for 1 h. Cells were then incubated with primary antibodies overnight. The primary antibodies included anti-GLUT1 (1:500, Abcam), anti-LDHA (1:250, Proteintech), and anti-HIF-1α (1:200, Abcam). The following morning, the cells were washed and incubated with CoraLite488-conjugated goat anti-rabbit/mouse IgG(H + L) (1:250, Proteintech) for 1 h. Cell climbing slices were then sealed with a mounting medium containing DAPI (Abcam, United States), and photographic images were acquired by confocal microscopy (Leica, Italy). Each experiment was repeated three independent times.

### 2.20 Immunohistofluorescence

After the measurement of hearing, mice were sacrificed and dislodged the encapsulated cochlea carefully. Dissected inner ears were fixed with 4% paraformaldehyde, decalcified in EDTA decalcifying solution, microdissected the cochlear epithelium, and then flatten the specimen to orient the sensory hair cell surface side up. Sections were blocked for 1 h with 10% goat serum. The primary antibody, anti-Myosin VIIa (1:50, Abcam) and 4-HNE (1:25, Abcam), anti-GLUT1 (1:500, Abcam), anti-LDHA (1:250, Proteintech), or anti-HIF-1α (1:100 Proteintech), were incubated overnight. Next day, sections were incubated with secondary antibodies for 1 h at room temperature: CoraLite594-conjugated goat anti-rabbit IgG(H+L) (1:250, Proteintech), CoraLite488-conjugated goat anti-mouse IgG(H+L) (1:250, Proteintech). Sections were sealed with mounting medium containing DAPI (Abcam, United States), and photographs were taken by a confocal microscopy (Leica, Italy).

### 2.21 Measurement of Hearing

6-week-old male C57 BL/6 mice (*n* = 10) were exposed to noise at a sound pressure level (SPL) of 110 dB for 2 h each day for two consecutive days. Under anesthesia (the continuous inhalation of 1.5% isoflurane), we recorded the auditory brainstem response (ABR) and distortion product otoacoustic emission (DPOAE) on a BioSigRP TDT System 3 (Tucker-Davis Technology, USA) to assess hearing thresholds before and after noise exposure. We used click and tone-pip ABRs to reflect the activity of the auditory nerve and the integrity of the auditory afferent pathway. DPOAE responses reflected the integrity of the outer hair cells and cochlear function.

### 2.22 Statistical Analysis

All data are shown as mean ± standard error of the mean (SEM). A two-tailed Student’s t-test was used to compare variables between two groups while one-way or two-way analysis of variance (ANOVA) was used to perform multi-group comparisons. Significant differences are represented by ns = not significant, *p < 0.05, **p < 0.01, and ***p < 0.001, and *p* values < 0.05 were considered to be statistically significant. Statistical details are included in the respective figure legends.

## 3 Results

### 3.1 t-BHP Exposure Induces Oxidative Stress Damage in HEI-OC1 Cells

**FIGURE 1:**
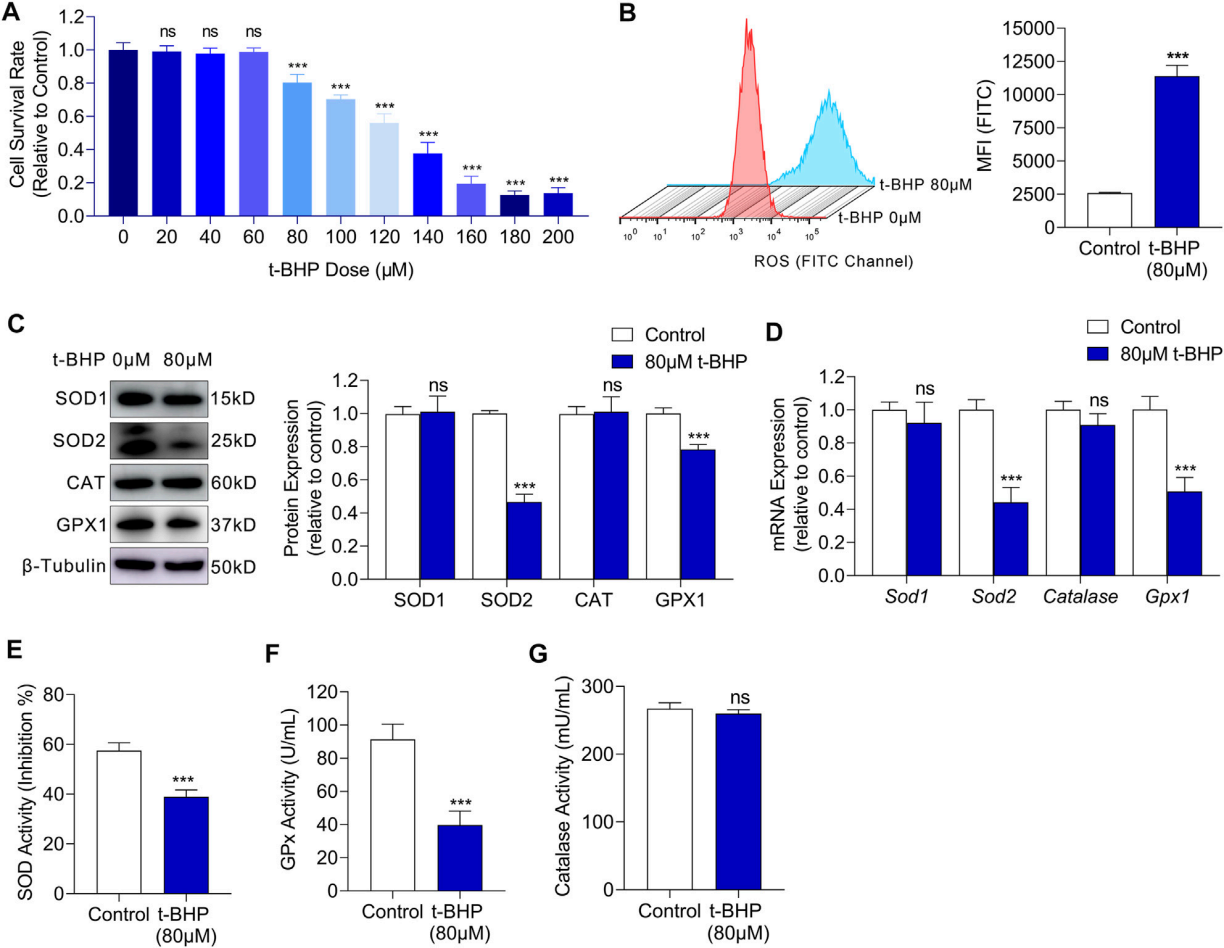
t-BHP exposure induced oxidative stress damage in HEI-OC1 cells. **(A)** HEI-OC1 cells were treated with gradient concentrations of t-BHP from 0 to 200 μM for 4 h. Cell viability was then determined CCK8 assays. **(B)** ROS levels of HEI-OC1 cells after treatment with 80 μM t-BHP for 4 h in comparison with controls. FITC fluorescence from a DCFH-DA probe was measured by flow cytometry. **(C)** Western blots showing the expression levels of ROS scavenging enzymes in HEI-OC1 cells treated with or without t-BHP. β-Tubulin was used as an internal reference. **(D)** RT-qPCR analysis of the genes encoding ROS scavenging enzymes in t-BHP treated HEI-OC1 cells relative to controls. *Tubb3* was used as an internal reference. **(E)** The enzymic activity of SOD in t-BHP-treated HEI-OC1 cells relative to controls, as determined by colorimetric analysis. **(F)** The enzymic activity of GPx in t-BHP-treated HEI-OC1 cells relative to controls, as determined by colorimetric analysis. **(G)** The enzymic activity of catalase in t-BHP-treated HEI-OC1 cells relative to controls, as determined by colorimetric analysis. Statistical results relate to mean MFI ± SEM. Data are represented as mean ± SEM. Bar charts were compared by the Student’s t-test or ANOVA (ns = not significant, *p < 0.05, **p < 0.01, and ***p < 0.001).

To investigate the glucose metabolism reprogramming in sensory hair cells under oxidative stress, we first established an *in vitro* oxidative stress injury model using HEI-OC1 cells. We cultured HEI-OC1 cells in different concentrations of t-BHP for 4 h and found that the cell viability decreased in a dose-dependent manner (Figure 1A). The treated HEI-OC1 cells exhibited a significant decrease in viability from a t-BHP concentration of 80 µM (Figure 1A), thus, this concentration was chosen for the following experiments. Previous reports have shown that the main cause of oxidative stress damage is the accumulation of ROS. Therefore, we measured the intracellular ROS levels in our cell model by flow cytometry. We found a markedly higher ROS level in HEI-OC1 cells treated with 80 µM t-BHP, compared with that of the control (Figure 1B). ROS accumulation implies that the intracellular ROS homeostasis is disrupted, hence we explored whether ROS clearance was impaired in the t-BHP-treated HEI-OC1 cells. Western blot and RT-qPCR analyses of ROS scavenging enzymes demonstrated that the expression of superoxide dismutase 2 (SOD2) and glutathione peroxidase (GPx) was markedly decreased after treatment with 80 µM t-BHP (Figures 1C,D). Additionally, the enzyme activity of SOD and GPx was significantly lower in the t-BHP-treated HEI-OC1 cells, compared with that in the control (Figures 1E–G). These observations suggest that the ability to scavenge ROS was inhibited by 80 µM t-BHP in HEI-OC1 cells. Taken together, t-BHP induced excessive ROS production and suppressed ROS removal, leading to intracellular ROS accumulation, which established a HEI-OC1 cell oxidative stress model.

### 3.2 Oxidative Stress Drives the Energy Metabolism Shift From Mitochondrial Oxidative Phosphorylation to Glycolysis in HEI-OC1 Cells

**FIGURE 2:**
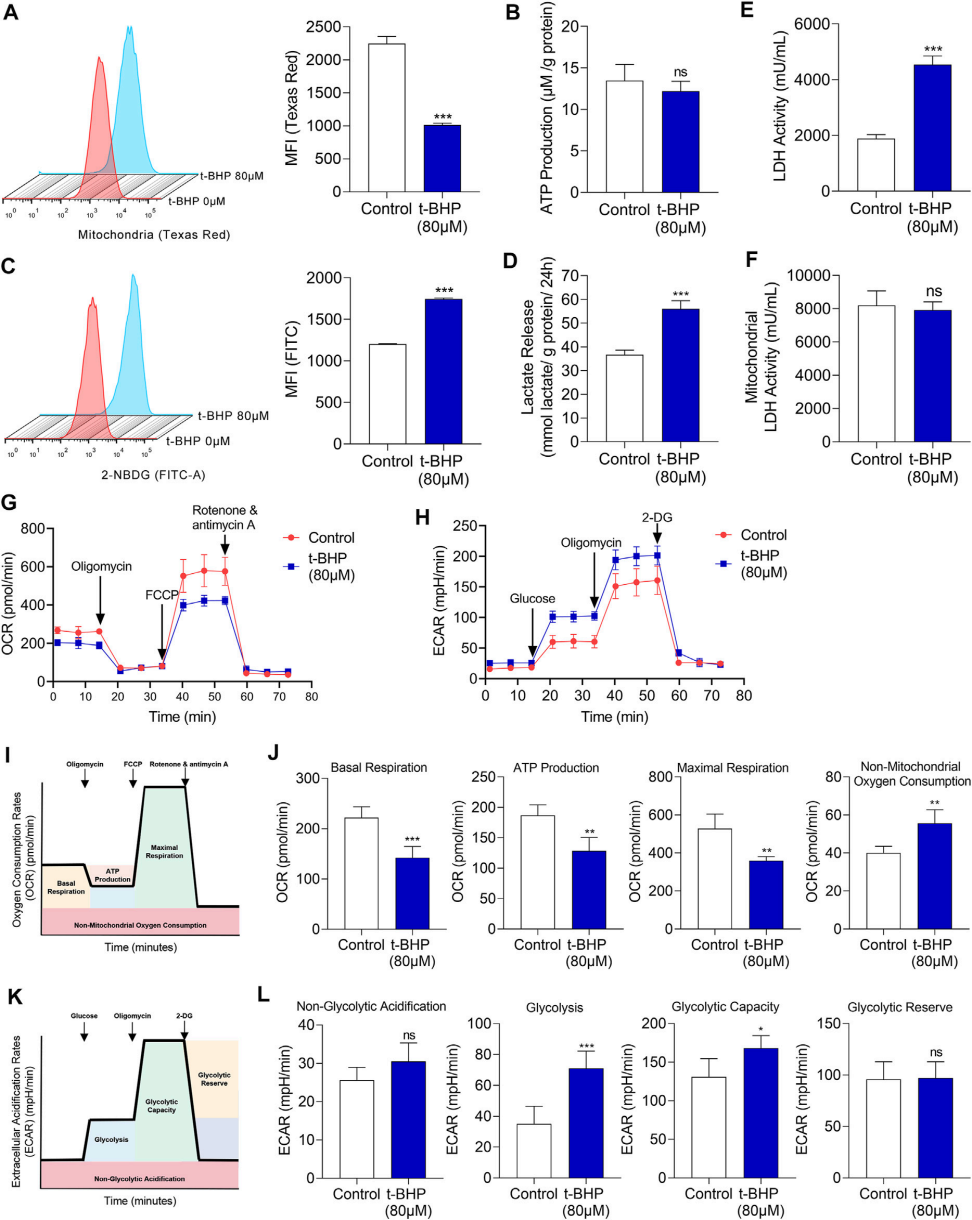
Glucose metabolic characteristics in t-BHP-treated HEI-OC1 cells. **(A)** The mitochondrial content of HEI-OC1 cells after treatment with 80 μM t-BHP for 4 h in comparison with controls. Texas Red fluorescence arising from Mito-tracker was measured by flow cytometry. **(B)** ATP production of t-BHP-treated HEI-OC1 cells relative to controls, as determined by fluorometric analysis. ATP production was normalized to total protein concentration. **(C)** 2-NBDG uptake by HEI-OC1 cells after treatment with 80 μM of t-BHP for 4 h in comparison with controls. FITC fluorescence derived from 2-NBDG was measured by flow cytometry. **(D)** Lactate release from t-BHP-treated HEI-OC1 cells compared to controls as determined by colorimetric analysis. Lactate levels were normalized to total protein concentration. **(E)** The enzymic activity of LDH in t-BHP-treated HEI-OC1 cells relative to controls, as determined by colorimetric analysis. **(F)** The enzymic activity of LDH in mitochondrial isolation of t-BHP-treated HEI-OC1 cells relative to controls, as determined by colorimetric analysis. **(G)** HEI-OC1 cells were seeded in a XFe96 cell culture plate. Next, we added 1.5 μM oligomycin, 1 μM FCCP, and 0.5 μM rotenone/antimycin A and monitored the OCR continuously. **(H)** HEI-OC1 cells were seeded in XFe96 cell culture plates. We then added 100 mM glucose, 10 μM oligomycin, and 500 mM 2-DG and monitored ECAR continuously. **(I)** The illustration of the typical OCR results. **(J)** A comparison of basal respiration, mitochondrial ATP production, maximal respiration, and non-mitochondrial oxygen consumption in t-BHP-treated HEI-OC1 cells relative to controls. **(K)** The illustration of the typical ECAR results. **(L)** A comparison of non-glycolytic acidification, basal glycolysis, glycolytic capacity, and glycolytic reserve, in t-BHP-treated HEI-OC1 cells relative to controls. Data are represented as mean ± SEM. Bar charts were compared by the Student’s t-test (ns = not significant, *p < 0.05, **p < 0.01, and ***p < 0.001).

To elucidate the metabolic characteristics of HEI-OC1 cells under oxidative stress, the changes of mitochondria were evaluated firstly. Mitochondria were labeled with fluorescent Mito-Tracker Texas Red and the mitochondrial contents were determined by flow cytometry. There was a significant decrease in the mitochondrial content in 80 µM t-BHP-treated HEI-OC1 cells compared with that in the control (Figure 2A). Sufficient supply of ATP is fundamental for the function of sensory hair cells (Wang and Puel 2018). Although 80 µM t-BHP induced a significant decrease in the mitochondrial content in HEI-OC1 cells, it did not reduce ATP production, compared with that of the control (Figure 2B). These findings suggest the existence of an alternative, mitochondria-independent energy supply pathway in HEI-OC1 cells under oxidative stress.

To reveal this alternative energy supply pathway, *in vitro* assays were performed on the HEI-OC1 cell oxidative stress injury model. We first analyzed the changes in energy substrates in HEI-OC1 cells treated with t-BHP. To measure the glucose uptake, we quantified 2-NBDG uptake. We observed a significant increase in glucose uptake in the 80 µM t-BHP-treated HEI-OC1 cells, compared with that in the control (Figure 2C). We also observed a markedly increased lactate release from the t-BHP-treated HEI-OC1 cells compared with that from the control cells (Figure 2D). In addition, LDH activity of whole cell lysates was significantly higher (Figure 2E) while LDH activity of mitochondrial isolates was no significant changes (Figure 2F) in the t-BHP-treated HEI-OC1 cells, compared with that in the control. Furthermore, OCR and ECAR were monitored continuously in our cell model. We successively added 1.5 µM oligomycin, 1 µM FCCP, and 0.5 µM rotenone/antimycin A and documented the changes in OCR. HEI-OC1 cells treated with 80 µM t-BHP demonstrated a significant decrease in OCR, compared with the control (Figure 2G). Furthermore, basal respiration, maximal respiration, and mitochondria-derived ATP production were significantly decreased in the HEI-OC1 cells under oxidative stress, compared with those in the control (Figure 2J). Next, the changes in ECAR were recorded after sequential addition of glucose (100 mM), oligomycin (10 µM), and 2-DG (500 mM). ECAR was significantly increased by t-BHP in HEI-OC1 cells (Figure 2H), and glycolysis and the glycolytic capacity increased in HEI-OC1 cells under oxidative stress, compared with those in the control (Figure 2L). Taken together, the increased lactate release and decreased oxygen consumption imply that glycolysis was possibly the alternative energy supply pathway that compensated for the reduced mitochondrial oxidative phosphorylation in HEI-OC1 cells under oxidative stress.

**FIGURE 3:**
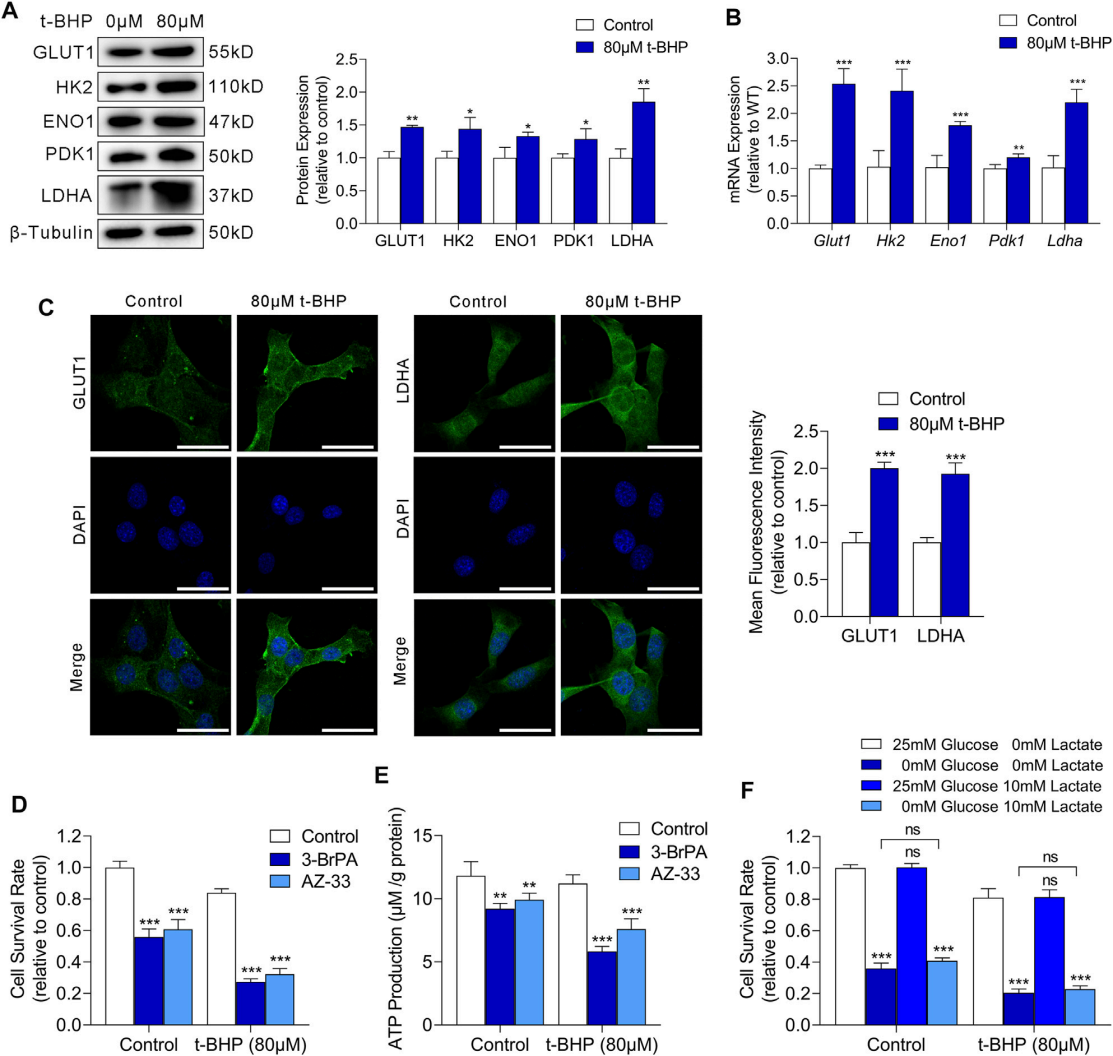
Oxidative stress drives glycolysis in HEI-OC1 cells. **(A)** Western blots of GLUT1 and the expression levels of glycolytic enzymes in HEI-OC1 cells treated with or without t-BHP. β-Tubulin was used as an internal reference. **(B)** RT-qPCR analysis of the expression levels of *Glut1* and glycolytic enzymes in t-BHP-treated HEI-OC1 cells relative to controls. *Tubb3* was used as an internal reference. **(C)** Immunocytofluorescence staining of GLUT1 and LDHA, the nuclei were stained blue with DAPI. Scale bar = 10 μm. **(D)** Cell viability was measured by CCK8 assays on treatment with or without 50 μM 3-BrPA or 2 μM AZ-33 for 48 h. HEI-OC1 cells cultured without 3-BrPA and AZ-33 were used as a baseline. **(E)** ATP production of 50 μM 3-BrPA or 2 μM AZ-33-treated HEI-OC1 cells relative to controls, as determined by fluorometric analysis. ATP production was normalized to total protein concentration. **(F)** Effect of glucose deprivation or exogenous lactate supplementation on the survival of HEI-OC1 cells. Different concentrations of glucose and lactate were added to the medium for 48 h. HEI-OC1 cells cultured in normal complete DMEM medium were used as a baseline. Data are represented as mean ± SEM. Bar charts were compared by the Student’s t-test (ns = not significant, *p < 0.05, **p < 0.01, and ***p < 0.001).

We next examined the mRNA and protein expression of glucose transporter 1 (GLUT1) and key glycolysis enzymes including hexokinase 2 (HK2), enolase 1 (ENO1), pyruvate kinase M2 (PKM2), and lactate dehydrogenase A (LDHA) in HEI-OC1 cells. We observed markedly increased expression of both GLUT1 and glycolytic enzymes in t-BHP-treated HEI-OC1 cells, compared with that in the control, at the protein and mRNA level (Figures 3A,B, respectively). Immunofluorescence images showed an increased membrane expression of GLUT1 and increased cytosolic expression of LDHA in HEI-OC1 cells under oxidative stress, compared with those in the control (Figure 3C).

To determine whether glycolysis is critical for HEI-OC1 cell survival under oxidative stress, we cultured HEI-OC1 cells with or without 80 µM t-BHP for 4 h, and then treated them with 3-BrPA (a selective HK2/glycolysis inhibitor) (Zhang et al., 2021) or AZ-33 (an effective lactate dehydrogenase A inhibitor) (Ward et al., 2012) for 48 h. Glycolytic inhibitors dramatically decreased cell survival (Figure 3D) and ATP production (Figure 3E) of t-BHP-treated HEI-OC1 cells compared to control cells. These findings confirm that the increase in glycolytic ATP production may play a compensatory role that contributes to the survival of t-BHP-treated HEI-OC1 cells.

Glucose and lactate are crucial carbon sources for cellular bioenergetic needs. To analyze the dependence of t-BHP-treated HEI-OC1 cells on glucose and lactate metabolism, we cultured HEI-OC1 cells with or without 80 µM t-BHP for 4 h, and then cultured them under low glucose (0.5 mM glucose) or exogenous lactate supplementation conditions (10 mM lactate) for 48 h. T-BHP-treated HEI-OC1 cells had higher glucose demand compared to the control (Figure 3F). Under low glucose conditions, lactate was not utilized as an alternative carbon source for cell survival in t-BHP-treated HEI-OC1 cells (Figure 3F).

### 3.3 Increased Glycolysis via HIF-1α Plays a Protective Role Against Oxidative Stress Damage in HEI-OC1 Cells

**FIGURE 4:**
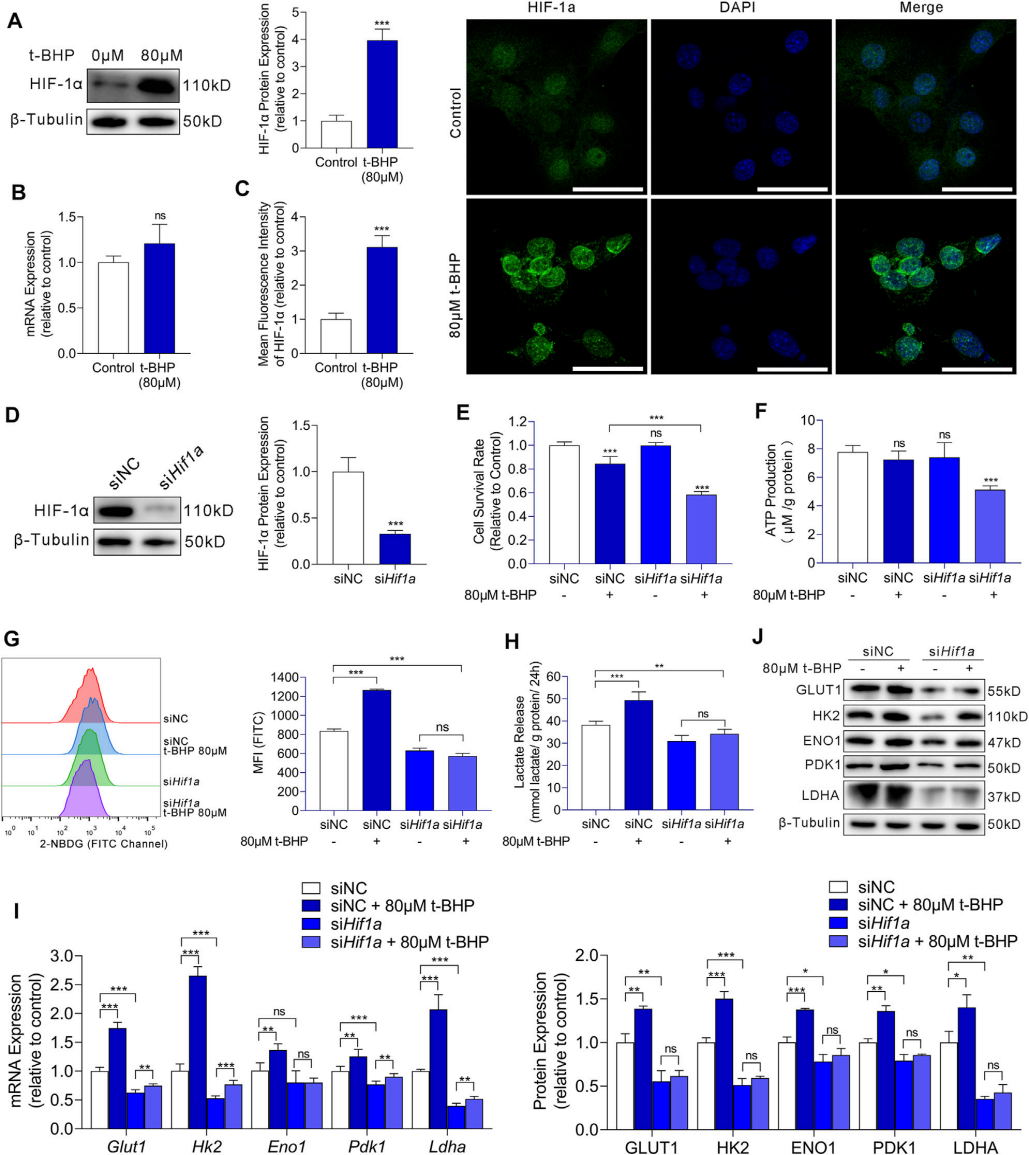
HIF-1α-induced glycolysis in response to oxidative stress in HEI-OC1 cells. **(A)** Western blots of HIF-1a expression in HEI-OC1 cells treated with or without t-BHP. β-Tubulin was used as an internal reference. **(B)** RT-qPCR analysis for the expression levels of Hif1a in t-BHP-treated HEI-OC1 cells relative to controls. *Tubb3* was used as an internal reference. **(C)** Immunocytofluorescence staining of HIF-1a (green); nuclei were stained blue with DAPI. Scale bar = 10 μm. **(D)** siRNA was used to knock down *Hif1a* expression levels in HEI-OC1 cells. The knockdown of *Hif1a* was confirmed by western blotting. β-Tubulin was used as a loading control. **(E)** The viabilities of HEI-OC1 cells transfected with siNC and si*Hif1a* and treated with or without 80 μM t-BHP were determined by CCK8 assays. **(F)** The ATP production by HEI-OC1 cells transfected with siNC and si*Hif1a* and treated with or without 80 μM t-BHP was determined by fluorometric analysis. ATP production was normalized to total protein concentration. **(G)** 2-NBDG uptake by HEI-OC1 cells transfected with siNC and si*Hif1a* and treated with or without 80 μM t-BHP was determined by flow cytometry. **(H)** Lactate release by HEI-OC1 cells transfected with siNC and si*Hif1a* and treated with or without 80 μM t-BHP was determined by colorimetric analysis. Lactate levels were normalized to total protein concentration. **(I)** RT-qPCR analysis of the expression levels of *Glut1* and glycolytic enzymes in HEI-OC1 cells transfected with siNC and si*Hif1a* and treated with or without 80 μM t-BHP. *Tubb3* was used as an internal reference. **(J)** Western blots of GLUT1 and the expression levels of glycolytic enzymes in HEI-OC1 cells transfected with siNC and siHif1a and treated with or without 80 μM t-BHP. β-Tubulin was used as an internal reference. Data are represented as mean ± SEM. Bar charts were compared by Student’s t-test or ANOVA (ns = not significant, *p < 0.05, **p < 0.01, and ***p < 0.001).

We demonstrated that compensatory energy production from increased glycolysis protected HEI-OC1 cells against oxidative stress injury in excessive ROS conditions. We then focused on the regulatory mechanism upstream of glycolysis in HEI-OC1 cells under oxidative stress. Studies have indicated that HIF-1α is the most important factor in the metabolic switch to glycolysis. Thus, we first examined the HIF-1α expression in our HEI-OC1 cell model by western blotting and RT-qPCR. Compared with that in the control, HIF-1α expression at the protein level was significantly higher in the 80 µM t-BHP-treated HEI-OC1 cells (Figure 4A), but there was no significant difference at the mRNA level (Figure 4B). Furthermore, immunofluorescence analysis showed that the HIF-1α expression in HEI-OC1 cells under ROS overload was higher than that in the control cells, and that HIF-1α protein localized in the nucleus (Figure 4C).

To further evaluate the effects of HIF-1α in HEI-OC1 cells on glycolysis and protection against oxidative stress, we knocked down HIF-1α with Hif1a-targeting siRNA (*siHif1a*) and compared the results with the corresponding control (siNC). Western blotting showed the Hif1a knockdown efficiency (Figure 4D).

Next, we treated siNC or siHif1a-HEI-OC1 cells with 80 µM t-BHP or saline for 4 h. A significant decrease in cell viability was observed in the si*Hif1a*-HEI-OC1 cells after treatment with t-BHP, compared with that in siNC-HEI-OC1 cells (Figure 4E). These results indicated that HIF-1α may contribute to the attenuation of ROS-induced sensory hair cell injury. Next, we determined the ATP production, 2-NBDG uptake, and lactate release in siNC or si*Hif1a*-HEI-OC1 cells treated with or without 80 µM t-BHP. Compared with that in the siNC-HEI-OC1 cells, a significant decrease in ATP production was observed in the si*Hif1a* -HEI-OC1 cells after t-BHP treatment (Figure 4F). Additionally, the 2-NBDG uptake and lactate release by the si*Hif1a*-HEI-OC1 cells were not affected by t-BHP (Figures 4G,H). Furthermore, there was no change in the expression of GLUT1 and glycolytic enzymes in the si*Hif1a*-HEI-OC1 cells with or without t-BHP treatment as assessed by RT-qPCR (Figure 4I) and western blotting (Figure 4J).

In summary, the activation of glycolysis by oxidative stress in HEI-OC1 cells was mediated by HIF-1α signaling. It is reasonable to argue that the HIF-1α-mediated energy supply compensation plays a potential protective role against oxidative stress damage in HEI-OC1 cells.

### 3.4 The Changes in the Oxidative Stress-HIF-1α-Glycolysis Axis in NIHL Mice

**FIGURE 5:**
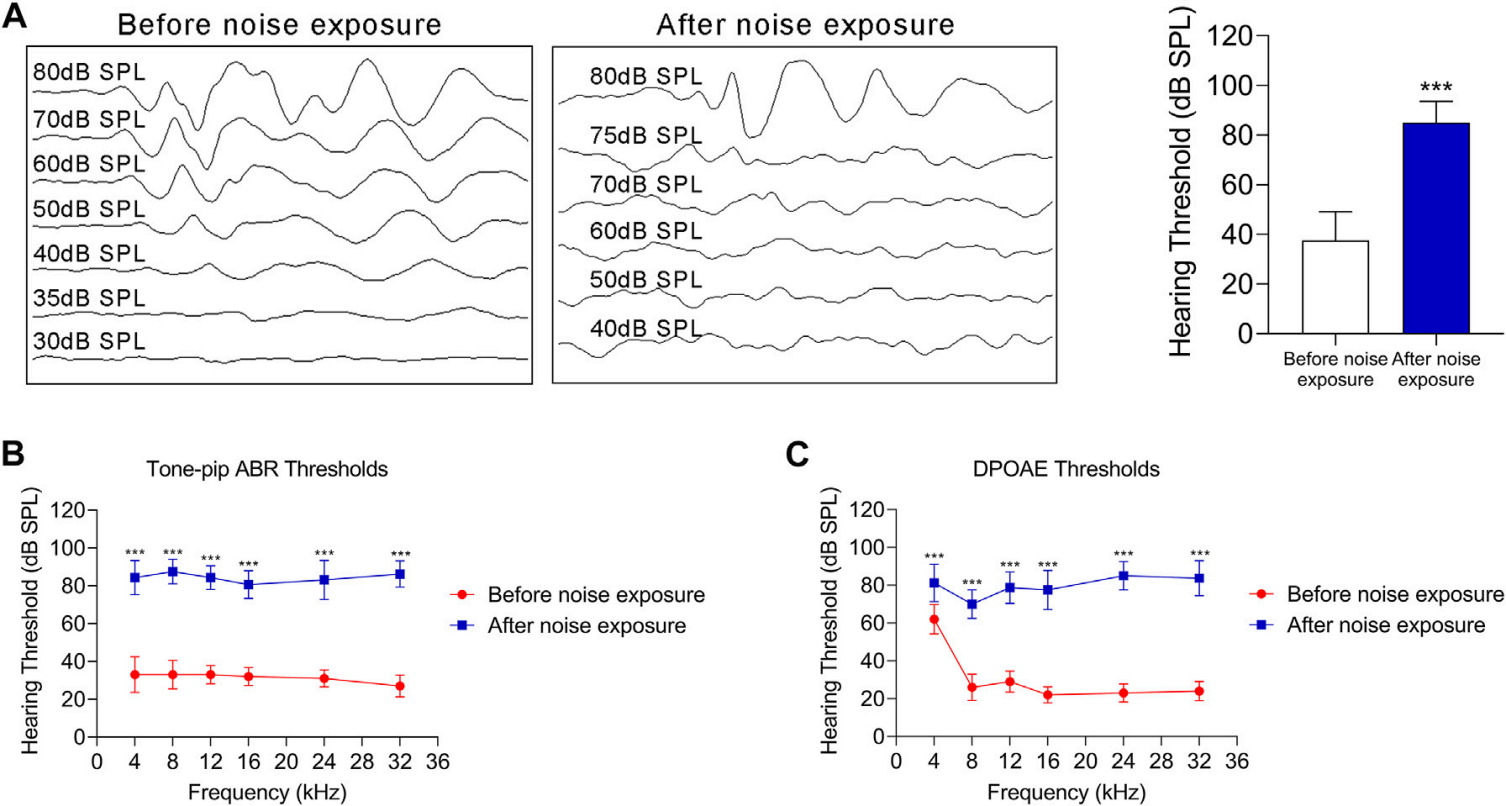
The NIHL C57 BL/6 mouse model. **(A)** ABRs thresholds in response to click stimulus in C57 BL/6 mice before and after noise exposure (110 dB SPL for 2 h each day for 2 consecutive day). Representative ABR recordings in response to click stimulus (left panel), and statistical results (right panel). **(B)** ABR thresholds in response to tone-pip stimuli (4, 8, 12, 16, 24, and 32 kHz) in C57 BL/6 mice before and after noise exposure (110 dB SPL for 2 h each day for 2 consecutive days). **(C)** The DPOAE thresholds in response to stimuli (4, 8, 12, 16, 24, and 32 kHz) in C57 BL/6 mice before and after noise exposure (110 dB SPL for 2 h each day for 2 consecutive days). For all studies *n* = 10. Data are represented as mean ± SEM. The data were compared by Student’s t-test (***p < 0.001).

To verify the response of the oxidative stress-HIF-1α-glycolysis axis to noise injury *in vivo*, we established a noise-exposure hair cell injury mouse model. Mice were exposed to noise at a 110 dB SPL for 2 h each day for 2 consecutive days. The thresholds of the click and tone-pip ABRs were significantly higher than the baseline (Figures 5A,B). Additionally, after noise exposure, the DPOAE threshold was significantly increased at 4, 8, 12, 16, 24, and 32 kHz, compared with that at the baseline (Figure 5C). The above results suggest that the NIHL mouse model was successfully established.

**FIGURE 6:**
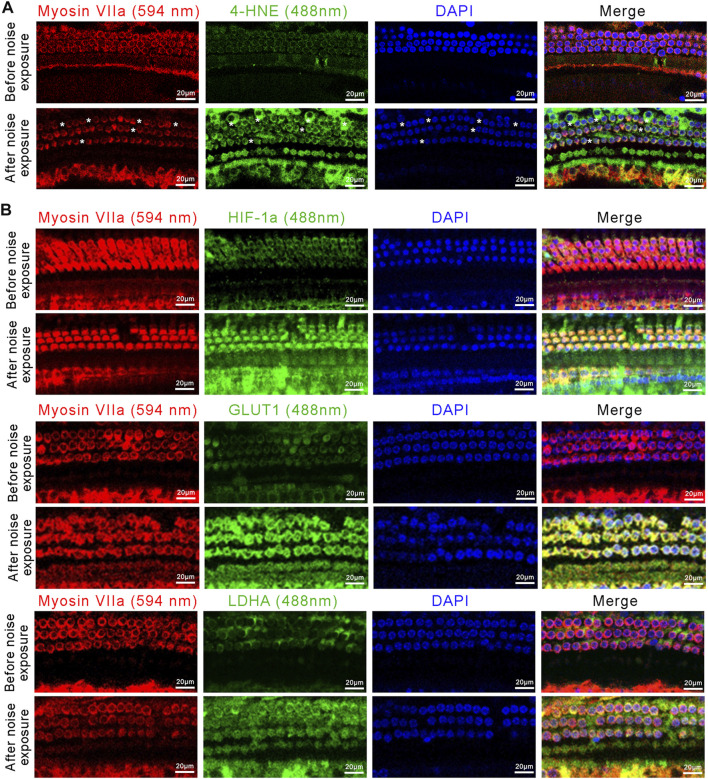
The activated oxidative stress-HIF-1α-glycolysis axis in NIHL mice. **(A)** Representative confocal images of the organ of Corti immunolabeled for Myosin VIIa (red) and 4-HNE (green), nuclei were stained blue by DAPI. Scale bar = 20 μm. Asterisks indicate the absence of outer hair cells. **(B)** Representative confocal images of the organ of Corti immunolabeled for Myosin VIIa (red) and HIF-1α, GLUT1, or LDHA (green), nuclei were stained blue by DAPI Scale bar = 20 μm.

We next assessed the changes in the oxidative stress-HIF-1α-glycolysis axis in the NIHL mouse model. We first examined the expression of 4-Hydroxynonenal (4-HNE), a major marker of oxidative stress, in outer hair cells. Immunofluorescence images showed high expression of 4-HNE after noise exposure, i.e., more pronounced signs of oxidative stress in outer hair cells after noise exposure (Figure 6A). Moreover, noise exposure resulted in significant loss of outer hair cells (Figure 5A). Immunofluorescence images also showed increased expression of HIF-1α, GLUT1, and LDHA in outer hair cells after noise exposure, compared with that in the control (Figure 6B). Taken together, enhanced oxidative stress, upregulated HIF-1α expression, and increased glycolytic enzymes’ expression were observed in NIHL mice, which is consistent with the results of the *in vitro* experiments on HEI-OC1 cells.

## 4 Discussion

To explore the roles of oxidative stress in the pathogenesis of NIHL, we established a t-BHP-induced oxidative stress damage model in HEI-OC1 cells and an NIHL model in mice. We demonstrated that noise trauma led to abnormal accumulation of ROS, which induced HIF-1α stabilization in sensory hair cells. HIF-1α as a key regulator of glycolysis, promoted GLUT1-mediated glucose uptake and upregulated the expression of glycolytic enzymes, which activated glycolysis in the ROS overloaded condition (Henderson et al., 2006; Wu et al., 2019). Our current study suggests that ROS-mediated upregulation of HIF-1α has a potential protective effect on sensory hair cells by reducing the damage of oxidative stress via remodeling glucose metabolism.

The sensory hair cell in the inner ear is a sensitive receptor for hearing and a converter responsible for the transformation of mechanical energy into an electrical signal (Yamashita et al., 1998). Sufficient energy supply is essential for sensory hair cells to work normally and effectively (Fuchs and Lauer 2019). Thus, efficient ATP production plays an important role in the function and fate of sensory hair cells (Butterfield and Halliwell 2019). ATP, the energy currency of the cell, is primarily produced by mitochondrial oxidative phosphorylation. Changes in mitochondrial structure and function are associated with the dysfunction and even death of sensory hair cells (He et al., 2017). This is consistent with our observations in HEI-OC1 cells after t-BHP treatment. Mitochondria as bio-engines also continually generate ROS as byproducts of the electron transport during oxidative phosphorylation, but under normal conditions this causes little damage, because the balance between ROS generation and ROS scavenging is a highly controlled process at homeostasis (Pan et al., 2019). Following noise exposure, sensory hair cells have an increased demand for ATP, which causes an increase in mitochondrial aerobic respiration. The excess mitochondrial ROS generation is due to the noise-induced intense metabolic activity in the sensory hair cells. This ROS overproduction may impair the redox balance, putting the sensory hair cells under ROS overload (Wu et al., 2020). Prolonged increased ROS generation will deplete ROS scavenging enzymes and exacerbate the ROS imbalance. Furthermore, the excess ROS will move into the cytosol and increase lipid peroxidation and superoxide production (Rogers et al., 2014). This will eventually directly damage mitochondrial membranes and lead to sensory hair cells death via apoptosis or necrosis. Therefore, we believe that mitochondrial dysfunction and ROS overload may be co-promoting glycolysis and may important pathological factors in the initiation and progression of NIHL.

Interestingly, we found that even though the mitochondrial content decreased with the increased ROS accumulation, the energy supply did not decrease in the sensory hair cells. A plausible explanation is that ROS accumulation resulted in mitochondrial impairment, manifesting as mitochondrial loss and reduced mitochondrial ATP synthesis, however, the ROS accumulation initiated one or more metabolic signaling pathways that compensated for the reduced mitochondrial ATP production (van Hameren et al., 2019). Our findings indicate that t-BHP-treated HEI-OC1 cells have increased 2-NBDG uptake, lactate release, extracellular acidification, glucose dependency, and decreased oxygen consumption, which reflected an increase in glycolytic energy production. Therefore, we suggest that energy metabolism shifts from mitochondrial oxidative phosphorylation to glycolysis in HEI-OC1 cells under oxidative stress. The present study suggests that ROS accumulation plays a role in the regulation of intracellular energy metabolism. Additionally, ROS accumulation triggers cellular adaptation and cellular energy supply preservation during oxidative stress, implying that it has a protective role. Accordingly, our preliminary findings suggest that there are complex roles for ROS in sensory hair cells after noise exposure, because it appears to have a dual role, which has a detrimental and protective effect on the pathophysiology of NIHL. This is likely to present a challenge for antioxidant therapy with ROS scavengers in NIHL. Therefore, in-depth studies are still required to further understand the molecular mechanisms of ROS in NIHL.

It has been suggested that ROS regulates signaling pathways in various physiological and pathophysiological processes, including adaptation to hypoxia, autophagy, immune adaptation, differentiation, and longevity (Finkel 2011; Collins et al., 2012; Sena and Chandel 2012). We focused on the interaction between ROS and HIF-1α. ROS contributes to the hypoxia-induced HIF-1α stabilization through the classical oxygen-dependent pathway (Niecknig et al., 2012; Zepeda et al., 2013). Additionally, ROS has a role in the oxygen-independent regulation to increase the stability of HIF-1α, and this non-classical pathway is thought to be related to the diminished PHDs activity and inhibited HIF-1α ubiquitin degradation (Semenza 2012). This is consistent with our observation that high HIF-1α expression was significantly induced by accumulated ROS in sensory hair cells after noise exposure. Previous studies on hearing loss have revealed that HIF-1α is a master regulator of the cellular adaptive response to hypoxia (Gross et al., 2003; Hwang et al., 2013). Our studies showed that increased HIF-1α was also a response to overload of intracellular ROS, therefore implicating a novel mechanism of HIF-1α-mediated protection of sensory hair cells during NIHL.

In the normal physiological state, the major source of ATP is mitochondrial oxidative phosphorylation (Fan et al., 2013). When mitochondrial function is damaged by excess ROS in sensory hair cells, compensatory glycolytic enhancement becomes an alternative pathway for generating ATP. Although the energy transformation of glycolysis is inefficient compared with that of oxidative phosphorylation, increased glycolysis still makes a significant contribution to cell survival under oxidative stress. HIF-1α is one of the most important regulatory factors of glycolysis, and this reprogramming of glucose metabolism in NIHL is mediated by HIF-1α (Semenza 2012). As a transcription factor, HIF-1α upregulates the expression of GLUT1 and a series of glycolytic enzymes (Gunton 2020). High glycolytic flux and increased glucose influx provide considerable ATP to maintain the function of sensory hair cells under oxidative stress. Although the outcome of sensory hair cells under excess ROS is irrecoverable, reprogramming of glucose metabolism plays an important role in the pathophysiological process of NIHL.

In the present study, we described a novel mechanism of HIF-1α-mediated protection of sensory hair cells during NIHL, which is triggered by overloaded intracellular ROS. We found that HIF-1α-induced glycolysis was activated by accumulated ROS when sensory hair cells are under increased oxidative stress in both *in vitro* and *in vivo* models. Moreover, we also demonstrated that upregulated HIF-1α enhanced the activation of glucose uptake and glycolysis, hence the energy supply was maintained by metabolic remodeling in the damaged sensory hair cells. Namely, activated glycolysis substituted the impaired mitochondrial oxidative phosphorylation to promote adaptation to the oxidative stress and improve sensory hair cells survival.

Under normal physiological conditions, cells maintain ROS homeostasis at a baseline level by controlling the balance between ROS production and clearance (Wang et al., 2019); whereas under stress conditions, ROS levels may fluctuate to alter signaling pathways (Tsutsumi et al., 2017). Previous studies have shown that ROS is an alarm system that is directly correlated with the amount of ROS produced and informs cells of changes in the extracellular environment (Knuppertz et al., 2017; Chen et al., 2020). The intensity of the stress that is beyond the cell tolerance induces larger quantities of ROS, which produces irreversible damage and subsequent death, while mild stress induces small amounts of ROS and does not lead to any serious consequences (Zhang et al., 2013; Sedlic et al., 2017). In sensory hair cells, ROS levels fluctuate over a wide range under various stress states, thereby causing diverse biological responses (Caprara et al., 2020). The precise role of ROS in sensory hair cells strongly depends on ROS levels. In the present study, we cultured HEI-OC1 cells with different concentrations of t-BHP which caused a dose-dependent decrease in cell viability at a t-BHP concentration of 80 µM. The lowest concentration capable of causing HEI-OC1 cell death and a 4–5-fold increase in intracellular ROS levels was 80 μM, and it was selected for subsequent experiments. Although the 80 µM t-BHP-treated HEI-OC1 cell model is well representative of oxidative stress injury, it cannot provide a comprehensive assessment of intracellular events. We have shown that ROS has a protective role against oxidative stress; however, considering the complexity of the roles of ROS, further in-depth *in vitro* and *in vivo* studies are needed.

In conclusion, our results demonstrated that accumulated ROS promoted remodeling of energy metabolism in sensory hair cells under oxidative stress via HIF-1α signaling. Therefore, ROS potentially has a protective role against noise-induced sensory hair cell damage.

## Data Availability

The original contributions presented in the study are included in the article/Supplementary Material, further inquiries can be directed to the corresponding author.
