# Supplementary material for: Accumulated ROS Activates HIF-1α-Induced Glycolysis and Exerts a Protective Effect on Sensory Hair Cells Against Noise-Induced Damage
Source: Front Mol Biosci. 2022 Jan 12;8:806650. doi: 10.3389/fmolb.2021.806650 (PMC8790562; doi:10.3389/fmolb.2021.806650)
Supplement: Supplementary file 4 [file DataSheet1.DOC]

Full scan images of Western Blots:

<https://www.jianguoyun.com/p/DW42sLAQt6f0CRjJ85YE>

Flow cytometry data:

<https://www.jianguoyun.com/p/DabAh5MQt6f0CRjF_JEE>
